# Vascular and autonomic correlates of screening-positive depressive symptom burden in chronic atrophic gastritis: a multicenter observational study protocol

**DOI:** 10.3389/fmed.2026.1842882

**Published:** 2026-05-05

**Authors:** Meng Wang, Shunlei Jiang, Zixiang Jin, Xinyu Pan, Shijie Xu, Chao Wang

**Affiliations:** 1 Institute of Basic Theory of Traditional Chinese Medicine, China Academy of Chinese Medical Sciences, Beijing, China; 2 Liyang Hospital of Chinese Medicine, Changzhou, China; 3 Department of Integrated Traditional Chinese and Western Medicine, Peking University First Hospital, Beijing, China; 4 Department of Endocrinology, Guang’anmen Hospital, China Academy of Chinese Medical Sciences, Beijing, China

**Keywords:** autonomic measures, chronic atrophic gastritis, gastroenterology outpatient clinics, observational study protocol, screening-positive depressive symptoms, vascular biomarkers

## Abstract

**Background:**

Depressive symptoms are common in gastroenterology practice but often missed due to patient stigma and the limitations of self-report questionnaires. This detection gap limits patient access to necessary mental health care. Although vascular dysfunction has been associated with depressive symptoms, the role of accessible peripheral vascular and autonomic measures in relation to concurrent questionnaire-defined depressive symptom burden in gastroenterology settings remains insufficiently studied. This study aims to evaluate whether vascular and autonomic measures are associated with screening-positive depressive symptom burden in chronic atrophic gastritis and whether they may provide adjunctive information beyond conventional clinical and histological variables.

**Methods and analysis:**

This multicenter observational protocol plans to enroll 450–520 adults aged 20–60 years with histologically confirmed chronic atrophic gastritis from four centers in China. The study includes dorsalis pedis artery Doppler ultrasonography, brachial–ankle pulse wave velocity assessment, gastrointestinal and histological evaluation, and standardized psychological questionnaires. The primary analysis examines whether vascular and autonomic measures are associated with concurrent screening-positive depressive symptom burden (PHQ-9 ≥ 10) and whether they provide incremental information beyond clinical and histological variables. Multivariable regression and internally validated discrimination analyses are used. No participants have been enrolled and no data have been collected at the time of submission.

**Discussion:**

The findings may help inform future validation studies and multidisciplinary assessment strategies in gastroenterology settings.

**Conclusion:**

This study aims to evaluate whether vascular and autonomic measures are associated with screening-positive depressive symptom burden in chronic atrophic gastritis and whether they may provide adjunctive information beyond conventional clinical and histological variables.

**Clinical trial registration:**

[ClinicalTrials.gov], identifier [NCT07319338].

## 1 Introduction

Chronic gastritis and related upper gastrointestinal disorders are common in clinical practice and often coexist with psychological symptoms such as depression and anxiety (1, 2), within a broader gut-brain framework that has attracted increasing attention (3–5). Within this broader group, chronic atrophic gastritis (CAG) is a chronic inflammatory gastric disorder characterized by glandular loss, with or without intestinal metaplasia, and is recognized as a premalignant condition in the Correa cascade. CAG is most commonly associated with Helicobacter pylori infection or autoimmune mechanisms and often requires long-term surveillance because of its pathological progression and cancer risk. We selected CAG as the target population because it provides a clinically and histologically defined gastroenterology setting in which persistent gastrointestinal burden, pathological severity, and psychological distress may coexist.

Even when psychological distress is clinically relevant, depressive symptoms may still be under-recognized in routine gastroenterology care. Patients who present mainly with somatic complaints may be less likely to describe emotional symptoms spontaneously, and self-report instruments alone may not fully capture distress in this setting. For this reason, it may be useful to explore adjunctive, non-psychiatric assessment approaches that can help clinicians open conversations about mental health in a way that feels more acceptable to patients.

Growing evidence suggests that vascular dysfunction and arterial stiffness are associated with depressive symptoms (6–8). In the present study, vascular and autonomic measures are not intended to explain the pathogenesis of CAG itself. Rather, they are being examined as accessible physiological measures that may be associated with concurrent depressive symptom burden within a clinically defined gastroenterology population. Brachial–ankle pulse wave velocity (baPWV) (9–11) and ankle–brachial index (ABI) (12) are established markers of vascular status, while Doppler ultrasound offers a practical way to assess peripheral hemodynamics in routine clinical settings (13). In this framework, gastrointestinal pathology provides the disease context, whereas dorsalis pedis artery Doppler indices, baPWV, and heart rate variability are evaluated as potential adjuncts to symptom-based assessment (14, 15). Dorsalis pedis ultrasound was selected primarily for pragmatic reasons, including superficial accessibility, feasibility in routine outpatient settings, and compatibility with standardized repeated measurement, rather than because of established biological specificity for depressive symptom burden. In the present protocol, it is therefore treated as an accessible peripheral hemodynamic marker within a multimodal assessment framework rather than as a uniquely informative vascular bed.

Currently, most studies in this area remain focused on symptom questionnaires (16), inflammatory or microbial markers (17, 18), or broader gut–brain frameworks without a specific emphasis on identifying depressive symptom burden in routine gastroenterology settings (19, 20). This leaves a practical gap in adjunctive assessment approaches that may be acceptable to patients who primarily seek help for somatic gastrointestinal complaints. In this context, vascular and autonomic measures may offer an additional source of clinically interpretable information that can complement, rather than replace, conventional psychological assessment.

Our interest in this question was also informed by preliminary observations from our earlier work in related gastroenterology populations, although those findings are not yet published and therefore are not presented here as formal supporting evidence.

Against this background, we designed a multicenter observational study in routine gastroenterology settings. The primary aims are to characterize multimodal phenotypes across PHQ-9-defined depressive symptom strata, examine cross-sectional associations between vascular/autonomic markers and concurrent depressive symptom burden, and assess whether these markers provide incremental information beyond clinical and histological variables. This protocol is intended as a phenotyping and association study rather than a mechanistic validation study.

## 2 Study objectives

(1) Characterize multimodal phenotypes across depressive symptom strata.
(2) Quantify independent associations between distal vascular markers and questionnaire-defined depressive and anxiety symptom burden with prespecified adjustment.
(3) Develop and internally validate a parsimonious multivariable model for identifying PHQ-9 screening-positive depressive symptom burden, and evaluate the incremental information provided by vascular and HRV markers beyond clinical and histological variables.
(4) Descriptively summarize selected workflow-related indicators, including assessment completion, time burden, and referral uptake, to inform future practice-oriented and validation-focused research.

## 3 Methods

### 3.1 Study design and setting

This is a multicenter observational study conducted in routine gastroenterology outpatient settings. The primary analytic framework is cross-sectional and focuses on concurrent depressive symptom burden at baseline. The study is designed to characterize multimodal phenotypes, examine associations between vascular markers and depressive symptoms, and develop an internally validated multivariable model for identifying PHQ-9 screening-positive depressive symptom burden within a histologically defined CAG population. The protocol is coordinated by the China Academy of Chinese Medical Sciences (lead center). At the time of submission, site activation has been completed for the China Academy of Chinese Medical Sciences and Hengshui Hospital of Traditional Chinese Medicine. Additional participating sites, including Beijing Anzhen Hospital and Liyang Hospital of Traditional Chinese Medicine, will begin recruitment only after completion of site-specific ethical approval and local activation procedures. The study design also allows for the potential inclusion of additional qualified centers during the recruitment phase to further enhance generalizability. Eligible adults with CAG are consecutively enrolled from gastroenterology outpatient clinics. We do not recruit from inpatient wards to maintain comparability with routine clinic screening workflows. All research procedures are conducted in accordance with a pre-established study protocol, which includes participant screening, informed consent acquisition, multimodal assessments, and data entry.

Author affiliations reflect institutional appointments and do not necessarily indicate site activation or participant recruitment responsibilities within the present protocol.

### 3.2 Participants

Eligible participants will be patients diagnosed with chronic atrophic gastritis (CAG) confirmed by histopathology following upper gastrointestinal endoscopy at one of the participating hospitals. All subjects will be screened and registered according to standardized criteria.

#### 3.2.1 Inclusion criteria

Participants must meet all of the following criteria to be included: (1) Age between 20 and 60 years, regardless of sex; (2) underwent upper GI endoscopy at one of the participating centers within the past 6 months, with histologically confirmed CAG, defined by glandular atrophy with or without intestinal metaplasia according to the Updated Sydney System and staged using OLGA/OLGIM when feasible; (3) presence of upper GI symptoms (e.g., epigastric discomfort, pain, bloating, belching, early satiety) is recorded; symptomatic status is not required for inclusion; (4) ability to comprehend and voluntarily complete psychological assessments (e.g., depression and anxiety scales) and multimodal vascular examinations (e.g., dorsalis pedis artery ultrasound); (5) willingness to provide written informed consent and allow the use of their clinical data and examination results for research purposes.

The age range of 20–60 years was chosen to reduce heterogeneity related to adolescent developmental status and advanced age-related vascular comorbidity, thereby improving comparability across multimodal physiological assessments in the intended outpatient screening population.

#### 3.2.2 Exclusion criteria

Participants will be excluded if they meet any of the following conditions: (1) History of major psychiatric disorders (e.g., schizophrenia, bipolar disorder), or currently experiencing an acute psychotic episode; (2) Recent (within 3 months) major cardiovascular or cerebrovascular events, such as acute myocardial infarction or stroke; (3) Documented severe peripheral artery disease or major anatomical abnormalities in the lower extremity vasculature—e.g., Fontaine stage III–IV peripheral arterial occlusive disease, diabetic foot with extensive ulcers or gangrene—that would preclude reliable dorsalis pedis artery ultrasound assessment; (4) Presence of severe hepatic or renal dysfunction (e.g., significantly reduced eGFR), or malignancy with a limited life expectancy; (5) Current use of systemic corticosteroids or potent immunosuppressants that may significantly alter vascular function or psychological status; (6) Pregnancy or lactation; (7) Any other condition deemed by the investigators to interfere with study participation (e.g., inability to cooperate with examinations or follow-up).

Psychiatric specialty care and/or psychotropic medication use will not be exclusionary. Medication type, dose (standardized daily dose when feasible), and duration will be recorded and adjusted for; sensitivity analyses will exclude moderate-to-high intensity regimens.

#### 3.2.3 Grouping strategy

At baseline, all enrolled participants will complete the 9-item Patient Health Questionnaire (PHQ-9) to assess the severity of depressive symptoms. Based on their total PHQ-9 scores, participants will be categorized into two groups:

(1) Screen-negative group: PHQ-9 total score < 10;
(2) Screen-positive group: PHQ-9 total score ≥ 10.

In secondary analyses, depressive symptom severity may also be categorized using standard PHQ-9 severity bands (e.g., 0–4/5–9/10–14/15–19/20–27) to explore dose–response patterns; the primary grouping remains PHQ-9 < 10 vs. ≥ 10.

Rationale for grouping: PHQ-9 ≥ 10 was selected as a commonly used threshold for screening-positive, clinically relevant depressive symptom burden. In the present study, it is used as a questionnaire-defined screening outcome rather than a diagnostic criterion for depressive disorder.

**FIGURE 1:**
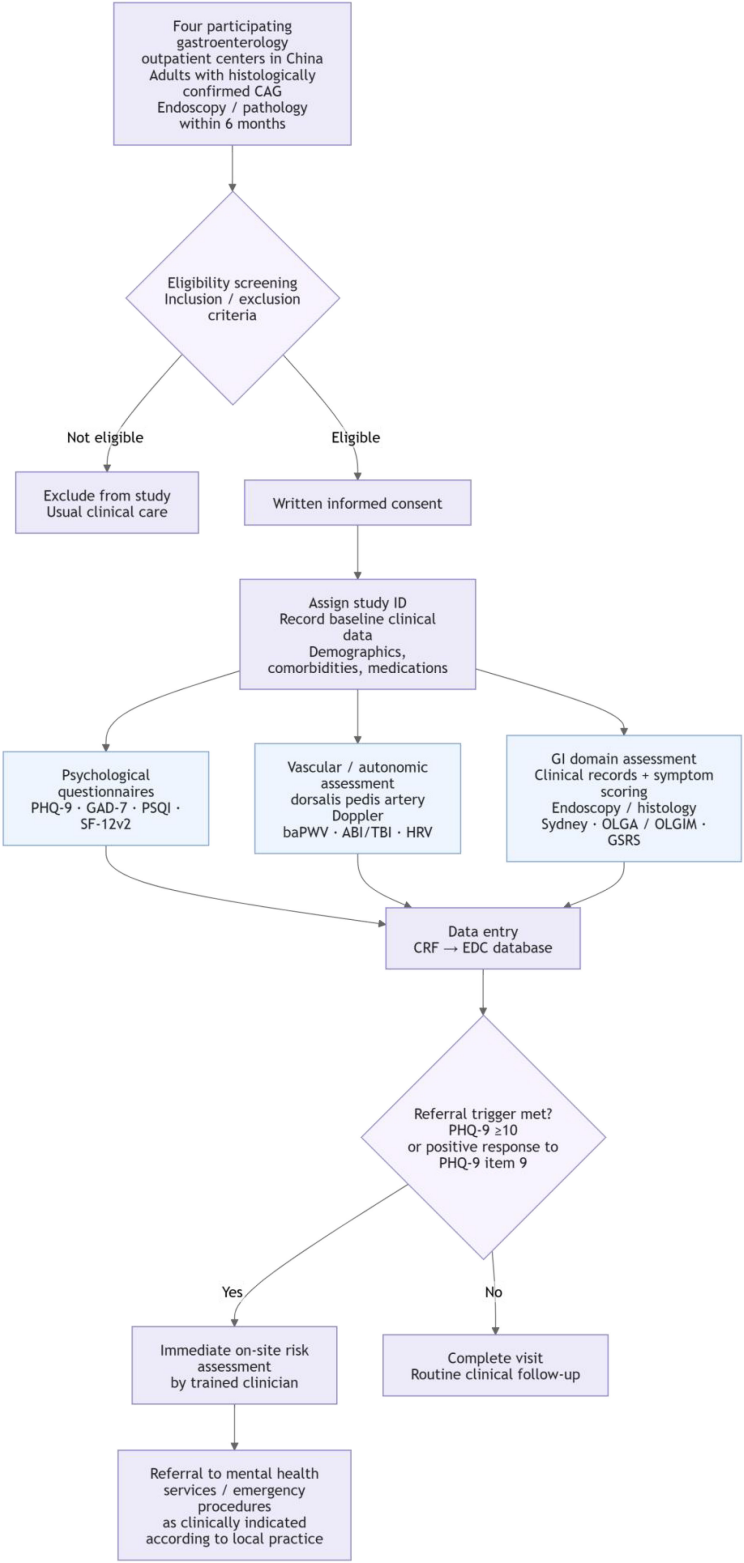
Flowchart of the multicenter study protocol. The workflow illustrates the pragmatic integration of objective vascular biomarkers (dorsalis pedis Doppler, baPWV, ABI/TBI) with routine psychological screening in gastroenterology clinics across four participating centers. Key steps: Eligibility screening → Informed consent → Multimodal data collection (questionnaires plus vascular/autonomic tests) → Cross-sectional classification of questionnaire-defined symptom burden → Routine clinical safety assessment when PHQ-9 ≥ 10 or PHQ-9 item 9 is positive. In the workflow, PHQ-9 ≥ 10 and any positive response to PHQ-9 item 9 function as combined clinical safety triggers for immediate assessment; analytically, however, the primary study outcome remains PHQ-9 ≥ 10, whereas item 9 positivity is used for safety management rather than as a co-primary endpoint.

The planned flow of patient screening, enrolment and multimodal assessment is summarized in Figure 1.

### 3.3 Multimodal data collection (study procedures)

All enrolled participants undergo a standardized multimodal evaluation protocol, comprising the following four primary domains. Adjusted covariates will include age, sex, body mass index, smoking, alcohol use, education level, marital status, hypertension, diabetes, dyslipidemia, Helicobacter pylori status, medication use, and OLGA/OLGIM stage.

#### 3.3.1 Gastrointestinal indicators

GI-related data include both objective findings (endoscopy and histopathology) and subjective symptom assessments.

##### 3.3.1.1 Endoscopic and histological grading

Gastric mucosal features such as inflammation, atrophy, and intestinal metaplasia are recorded according to the hospital’s standardized endoscopic and pathological diagnostic procedures. Histological grading follows the Updated Sydney System, with further staging using the OLGA/OLGIM systems when feasible, to quantify gastritis severity as ordinal categorical variables.

##### 3.3.1.2 GI symptom scoring

GI symptom severity is assessed using the Gastrointestinal Symptom Rating Scale (GSRS, 15 items, seven-point Likert), generating a total score and subdomain scores (e.g., abdominal pain, reflux, indigestion, diarrhea, constipation). GSRS will serve as the primary GI symptom measure for all analyses. Higher scores indicate worse symptoms.

##### 3.3.1.3 *Helicobacter pylori* status and eradication history

*H. pylori* status is obtained from routine clinical records within the same endoscopy episode or within 6 months, prioritizing histology and/or rapid urease test when available; alternatively, 13C/14C urea breath test or stool antigen tests will be accepted if performed in routine care. Status will be coded as positive/negative/unknown. Prior *H. pylori* eradication therapy (yes/no, approximate timing) is recorded where feasible and considered in adjustment/sensitivity analyses.

Given that histopathological changes such as atrophy and metaplasia progress slowly, prior studies suggest OLGA/OLGIM staging is relatively stable over a 6-month window. Thus, endoscopy/pathology performed within 6 months will be considered a valid “slow-varying” proxy of current gastric mucosal status. The interval between endoscopy/histopathology and multimodal assessment will be recorded as a continuous timing variable. In addition to a sensitivity analysis restricted to participants with an interval of ≤3 months, we will explore whether this interval materially influences association estimates.

#### 3.3.2 Peripheral vascular ultrasound and arterial stiffness metrics

Peripheral vascular status is assessed via dorsalis pedis artery ultrasound and standard arterial stiffness parameters.

##### 3.3.2.1 Dorsalis pedis artery ultrasound

Doppler angle correction (e.g., ≤60°) and sample volume placement are standardized across participants; at least three cardiac cycles are averaged for PSV/EDV-derived indices. Using B-mode imaging, the dorsalis pedis artery is visualized between extensor hallucis longus and extensor digitorum longus tendons. Measurements include arterial diameter and anatomic trajectory. Color and pulsed Doppler modes are then used to capture the full cardiac cycle waveform and derive:

① Peak systolic velocity (PSV)

② End-diastolic velocity (EDV)

③ Resistive index (RI = (PSV–EDV)/PSV)

④ Pulsatility index (PI = (PSV–EDV)/Vmean)

⑤ Flow waveform classification (triphasic, biphasic, or monophasic)

If feasible, vessel cross-sectional area and flow volume may also be estimated. By default, the right dorsalis pedis artery is assessed. If prior vascular surgery, deformities, or measurement constraints exist, the left side is used and documented. Bilateral measurements may be obtained in some cases to allow averaging or sensitivity analyses.

Consistency will be maximized by a harmonized acquisition protocol (probe type and frequency range, Doppler angle ≤ 60°, sample volume placement, wall filter/PRF, gain, sweep speed, and averaging over ≥ 3 cardiac cycles) with site-specific standardized presets and cross-site training. Where identical equipment is not feasible, the device model, transducer, and key presets will be recorded, and site/device effects will be explored in sensitivity analyses (and/or adjusted as covariates). Sonographers will be blinded to participants’ psychological scale scores and group assignments to minimize observer bias. Doppler images and waveform recordings will be centrally archived. A blinded secondary review of a randomly selected subset is performed by the lead center to assess cross-site consistency, and site/device effects will be examined analytically as sensitivity analyses. Waveform classification (triphasic, biphasic, or monophasic) will be analyzed as an ordinal categorical variable; in the blinded secondary review subset, any classification discrepancies will be resolved by adjudication at the lead center according to the prespecified waveform criteria.

To ensure inter-center consistency, a centralized training program is conducted by the lead center (Beijing). All participating sonographers undergo standard operating procedure (SOP) training and pass a consistency evaluation (Kappa > 0.8) before study commencement.

##### 3.3.2.2 Standard arterial stiffness measures

Within the same visit or within 2 weeks, participants undergo baPWV and ABI measurement using validated devices. Toe–brachial index (TBI) is also assessed when feasible. Participants must rest supine for 5–10 min prior to measurement, and procedures follow standardized device protocols and consensus guidelines to ensure reproducibility.

#### 3.3.3 Autonomic and physiological parameters

This domain includes:

##### 3.3.3.1 Blood pressure and heart rate

Resting seated or supine measurements are taken using an automated monitor, repeated 2–3 times with the average recorded for systolic/diastolic BP and resting HR.

##### 3.3.3.2 HRV

Short-term (5-min) HRV is recorded in a quiet, appropriately lit setting using a 3-lead ECG or RR interval–capable device. Recordings will be obtained during spontaneous breathing under standardized posture (supine or seated), with participants resting quietly throughout the procedure. Respiratory rate will be documented when feasible, and residual variation related to breathing pattern will be acknowledged as a potential limitation in interpretation. Accordingly, HRV-related findings will be interpreted cautiously in view of the potential residual confounding introduced by uncontrolled respiratory variation. Device sampling specifications will also be documented. R-wave detection and artifact removal are performed using validated software, and time-domain metrics are calculated, including:

① SDNN (standard deviation of normal-to-normal intervals).

② RMSSD (root mean square of successive differences).

③ Frequency-domain analyses may also be performed if applicable.

##### 3.3.3.3 Current medication use:

Information on the use of antihypertensive agents (including β-blockers), lipid-lowering agents, antiplatelet or anticoagulant drugs, and psychotropic medications (e.g., antidepressants, anxiolytics, hypnotics) prescribed in any setting (including psychiatric services) is systematically collected from medical records and patient interviews, and coded for adjustment; type, dose (standardized daily dose when feasible), and duration are recorded, and sensitivity analyses excluding moderate-to-high intensity regimens are performed.

These factors will be treated as potential sources of physiological confounding and addressed through covariate adjustment and prespecified sensitivity analyses where feasible.

#### 3.3.4 Psychological and symptom scales

Validated self-report instruments are used to assess psychological burden:

##### 3.3.4.1 Depression

The Patient Health Questionnaire-9 (PHQ-9) assesses depressive symptom severity over the past 2 weeks (total score: 0–27).

##### 3.3.4.2 Anxiety

The Generalized Anxiety Disorder-7 (GAD-7) assesses anxiety symptoms (total score: 0–21).

##### 3.3.4.3 Sleep quality

The Pittsburgh Sleep Quality Index (PSQI) assesses subjective sleep quality over the past month.

##### 3.3.4.4 Health-related quality of life

Health-related quality of life is measured using the SF-12v2, yielding Physical Component Summary (PCS) and Mental Component Summary (MCS) scores.

All questionnaires are administered in a quiet environment under trained staff guidance. For participants with low literacy, items will be read neutrally and responses recorded appropriately.

Safety and referral procedures: Participants with PHQ-9 ≥ 10 or any positive response to PHQ-9 item 9 will receive prompt on-site assessment by a trained clinician, with urgent referral or emergency procedures initiated when clinically indicated.

To promote consistency in post-screen communication, clinicians will use neutral, non-diagnostic language when discussing questionnaire results and referral recommendations.

This communication approach is included as part of a standardized post-screening referral procedure for safety and consistency of clinical communication, rather than as an intervention being formally evaluated in the present study.

This strategy is intended to standardize post-screen communication across sites and reduce unnecessary variation in routine clinical explanation. Referral uptake will therefore be summarized descriptively and not interpreted as an effect of vascular findings alone.

#### 3.3.5 Timing and reproducibility assessments

To minimize timing-related confounders, most assessments are performed during the same clinical episode. Endoscopy/histopathology must be within 6 months; other measures (ultrasound, baPWV/ABI/TBI, HRV, questionnaires) are completed within 2 weeks, ideally during a morning visit after an overnight fast of at least 8 h and at least 30 min without smoking, caffeine, or vigorous physical activity; when fasting is not feasible, the postprandial interval and recent caffeine/smoking will be recorded and considered in sensitivity analyses.

To assess reproducibility of dorsalis pedis ultrasound, an approximately 10% subset of participants (or at least 50 participants, depending on recruitment feasibility) undergoes repeat assessment. Intra-rater reliability is evaluated by repeat scanning after 1–2 weeks by the same sonographer, and inter-rater reliability by independent scanning by a second sonographer. Intraclass correlation coefficients (ICCs) are used for continuous Doppler indices, with ICC ≥ 0.75 prespecified as acceptable repeatability.

### 3.4 Study outcomes

#### 3.4.1 Primary outcome

Screening-positive depressive symptom burden, defined as PHQ-9 ≥ 10. This outcome indicates at least moderate questionnaire-defined depressive symptom burden and is intended to support further clinical assessment rather than establish a diagnosis of depressive disorder.

No structured psychiatric interview or clinician-confirmed diagnostic reference standard is incorporated into the present protocol; accordingly, the primary outcome is a questionnaire-defined screening construct.

#### 3.4.2 Secondary outcomes

① PHQ-9 total score as a continuous variable (gradient of depression severity);

② GAD-7 total score (anxiety severity);

③ PSQI total and subscale scores (subjective sleep quality);

④ Descriptive workflow indicators: To characterize selected practical aspects of the assessment process in routine gastroenterology settings, we summarize:

Screening completion rate: The proportion of eligible patients who consent to and complete the vascular and psychological assessment.

Referral uptake: The proportion of screen-positive patients (PHQ-9 ≥ 10) who complete a mental health consultation within 4 weeks, as verified by medical records.

Time burden: The median time added to the routine gastroenterology workflow per patient.

### 3.5 Statistical analysis

Statistical analyses are prespecified using standard software (e.g., SPSS, R, or Stata). Two-sided tests are used with a significance level of α = 0.05.

#### 3.5.1 Descriptive statistics

Statistical analyses are prespecified using standard software (e.g., SPSS, R, or Stata). Continuous variables are expressed as mean ± SD or median (IQR), depending on distribution. The clinic-based prevalence of PHQ-9 ≥ 10 is estimated with 95% confidence intervals. We also summarize selected workflow-related indicators, including completion rates of questionnaires and vascular measurements and the proportion of screen-positive participants who complete referral when indicated. These measures are summarized descriptively [proportions with 95% CIs; time burden as median (IQR)] without hypothesis testing.

#### 3.5.2 Group comparisons

Participants will be grouped according to the primary outcome definition (PHQ-9 ≥ 10 vs. < 10). Between-group comparisons will evaluate differences in study indicators [gastric histopathology, vascular indices (e.g., RI, PI), baPWV, ABI/TBI, HRV, and scale scores] between PHQ-9 screening groups (≥0 vs. <10), while baseline demographics/clinical characteristics will be presented descriptively (Table 1). Comparisons of multimodal vascular, autonomic, and psychiatric measures between PHQ-9 screening groups (≥10 vs. <10) will be presented in Table 2.

**TABLE 1:** Baseline characteristics of the study participants according to depressive symptom status.

Variable	Total (*n* = …)	PHQ-9 < 10 (*n* = …)	PHQ-9 ≥ 10 (*n* = …)	SMDs
Demographics				
Age, years				
Female, *n* (%)				
Body mass index, kg/m^2^				
Lifestyle and comorbidities				
Current smoker, *n* (%)				
Alcohol use, *n* (%)				
Hypertension, *n* (%)				
Diabetes mellitus, *n* (%)				
Dyslipidaemia, *n* (%)				
History of cardiovascular disease, *n* (%)				
Gastric/endoscopic–histological features				
OLGA stage 0–II, *n* (%)				
OLGA stage III–IV, *n* (%)				
OLGIM stage 0–II, *n* (%)				
OLGIM stage III–IV, *n* (%)				
*H. pylori* positive, *n* (%)				
Psychiatric and symptom scales				
GAD-7 total score				
PSQI total score				
GSRS total score (and subscales if used)				
HRQoL physical component score				
HRQoL mental component score				

**TABLE 2:** Multimodal vascular, autonomic and psychiatric profiles according to depressive symptom status.

Variable	PHQ-9 < 10 (*n* = …)	PHQ-9 ≥ 10 (*n* = …)
Peripheral vascular ultrasound (dorsalis pedis artery)		
Inner diameter (mm)		
PSV (cm/s)		
EDV (cm/s)		
RI		
PI		
Triphasic waveform, *n* (%)		
Biphasic waveform, *n* (%)		
Monophasic waveform, *n* (%)		
Arterial stiffness and peripheral artery indices		
baPWV (cm/s)		
ABI		
TBI (if available)		
Autonomic and physiological measures		
Systolic blood pressure (mmHg)		
Diastolic blood pressure (mmHg)		
Resting heart rate (bpm)		
SDNN (ms)		
*RMSSD (ms)*		
Psychiatric and symptom measures		
GAD-7 total score		
PSQI total score		
GSRS total score (and subscales if used)		
HRQoL physical component score		
HRQoL mental component score		

Baseline characteristics are summarized descriptively and quantified using standardized mean differences (SMDs) rather than hypothesis testing; *p*-values will not be reported for Table 1. Formal statistical testing is used for prespecified exposure–outcome comparisons and model estimation as described below:

① *t*-test for normally distributed continuous variables with equal variances;

② Mann–Whitney U for non-normal or heteroscedastic variables;

③ χ^2^ test or Fisher’s exact test for categorical data.

#### 3.5.3 Multivariable models

Logistic regression will assess the independent association between key vascular markers (e.g., dorsalis pedis RI, PI, waveform classification, baPWV, ABI/TBI) and PHQ-9 ≥ 10, adjusting for study center (as a fixed effect), age, sex, body mass index, smoking status, alcohol use, education level, marital status, hypertension, diabetes, dyslipidemia, relevant medication use, and histopathological severity (e.g., OLGA/OLGIM stage or Updated Sydney graded atrophy/intestinal metaplasia). Results will be reported as odds ratios (OR) with 95% confidence intervals (CI). We will restrict the number of predictors in the primary multivariable models to approximately 10–12, prioritizing predefined key vascular indices and major confounders, with additional variables explored only in sensitivity analyses.

In a secondary analysis, linear regression will evaluate the association between vascular indices and PHQ-9 as a continuous outcome. Prior to model fitting, collinearity among vascular indices (e.g., RI, PI, baPWV, ABI/TBI) will be assessed (e.g., via variance inflation factors); if substantial collinearity is detected, we will reduce the number of simultaneously entered vascular predictors or construct composite scores to improve model stability. Model assumptions will be checked; transformations or generalized linear models will be applied as needed. Continuous predictors will be modeled linearly in the primary analysis unless clear evidence of non-linearity is identified; if linearity assumptions appear violated, appropriate transformations or restricted spline terms will be explored in secondary analyses. The results of the prespecified logistic and linear regression analyses will be presented in Table 3.

**TABLE 3:** Multivariable associations between vascular measures and depressive symptoms.

Table 3A | Logistic regression (outcome: PHQ-9 ≥ 10).			
Predictor	Model	OR (95% CI)	*P*-value
Dorsalis pedis RI (per 0.1 increase)	Model 1 (basic)		
Dorsalis pedis RI (per 0.1 increase)	Model 2 (extended)		
Dorsalis pedis PI (per 0.1 increase)	Model 1 (basic)		
Dorsalis pedis PI (per 0.1 increase)	Model 2 (extended)		
Monophasic vs. triphasic waveform	Model 2 (extended)		
baPWV (per 100 cm/s increase)	Model 2 (extended)		
ABI (per 0.1 increase)	Model 2 (extended)		
SDNN (per 10 ms increase)	Model 3 (with HRV)		
RMSSD (per 10 ms increase)	Model 3 (with HRV)		
Age (per 10-year increase)	All models (adjusted)		
Female sex	All models (adjusted)		
BMI (per 1 kg/m^2^ increase)	All models (adjusted)		
Hypertension	All models (adjusted)		
Diabetes mellitus	All models (adjusted)		
Dyslipidaemia	All models (adjusted)	–	–
OLGA stage III/IV vs. 0–II	All models (adjusted)	–	–
Model 1: adjusted for age, sex, body mass index (BMI), and conventional cardiovascular risk factors. Model 2: Model 1 plus histological severity of gastritis and standard arterial stiffness/peripheral artery indices (baPWV and ABI/TBI). Model 3: Model 2 plus heart rate variability (HRV) indices.			
**Table 3B | Linear regression (outcome: PHQ-9 total score).**			
**Predictor**	**Model**	**β (SE)**	***P*-value**
Dorsalis pedis RI (per 0.1 increase)	Model A (basic)		
Dorsalis pedis PI (per 0.1 increase)	Model A (basic)		
Monophasic vs. triphasic waveform	Model B (extended vascular)		
baPWV (per 100 cm/s increase)	Model B (extended vascular)		
ABI (per 0.1 increase)	Model B (extended vascular)		
SDNN (per 10 ms increase)	Model C (with HRV)		
RMSSD (per 10 ms increase)	Model C (with HRV)		
Age (per 10-year increase)	All models (adjusted)		
Female sex	All models (adjusted)		
BMI (per 1 kg/m^2^ increase)	All models (adjusted)		
Hypertension	All models (adjusted)		
Diabetes mellitus	All models (adjusted)		
Dyslipidaemia	All models (adjusted)		
OLGA stage III/IV vs. 0–II	All models (adjusted)		

#### 3.5.4 Incremental value analysis

Three hierarchical models for identifying PHQ-9 ≥ 10 at baseline will be compared:

① Model A (Clinical-GI Model): Demographics + cardiovascular risk factors + gastritis severity

② Model B (Vascular Multimodal Model): Model A + dorsalis pedis ultrasound + baPWV/ABI/TBI

③ Model C (Extended Multimodal Model): Model B + HRV metrics (e.g., SDNN, RMSSD)

Area under the ROC curve (AUC) and 95% CI will compare model performance. Among model-performance measures, AUC will be treated as the primary discrimination metric, whereas calibration indices, Brier score, and decision-curve analysis will be considered supplementary or secondary performance assessments. Model calibration will be assessed using calibration plots and, where appropriate, calibration slope/intercept and Brier score. We will additionally consider decision-curve analysis to evaluate potential clinical utility across plausible referral thresholds.

The primary inferential analysis will be a prespecified multivariable logistic regression model for PHQ-9 ≥ 10 including a limited set of predefined vascular exposures and major confounders. Linear models for PHQ-9 total score, penalized regression, composite-score approaches, and decision-curve analysis will be considered secondary or exploratory analyses. In the primary model, vascular predictors will be limited to a small prespecified set of clinically interpretable measures, such as dorsalis pedis RI and/or PI together with baPWV, while additional vascular or autonomic variables will be evaluated only in secondary or exploratory analyses.

### 3.6 Sample size and effect size estimation

The primary outcome is concurrent depressive symptom burden defined as PHQ-9 ≥ 10. The primary exposures of interest are dorsalis pedis artery Doppler indices, including resistive index and pulsatility index, together with arterial stiffness measures such as brachial–ankle pulse wave velocity.

Previous studies in populations with functional dyspepsia or chronic gastritis have reported a prevalence of PHQ-9 ≥ 10 in approximately 20%–30%. Although specific data in CAG are limited, we conservatively assume a similar prevalence, and the expected prevalence of moderate-to-severe depressive symptoms in our study sample is estimated at 25%.

To ensure model stability in multivariable logistic regression, we adopt the empirical rule of at least 10 events per variable (EPV ≥ 10). This sample size also permits estimating the prevalence of PHQ-9 ≥ 10 with acceptable precision (95% CI) in clinic-based CAG populations. Given that we plan to include approximately 10–12 covariates (including key vascular exposures and confounders), a minimum of 100–120 events (i.e., participants with PHQ-9 ≥ 10) is required. Assuming a 25% event rate, this translates to a total sample size of approximately 400–480 participants. Accounting for potential 10% missing data or incomplete assessments (e.g., partial vascular/HRV measurements), the target enrollment is set at 450–520 participants. Missingness will be summarized by variable and mechanism will be explored. Multiple imputation by chained equations (MICE) is used for covariates (and exposures if missingness is non-negligible), with the outcome included in the imputation model. Primary analyses report MI estimates; complete-case analyses are presented as sensitivity analyses.

If the observed prevalence of PHQ-9 ≥ 10 is substantially lower than anticipated and the number of events is insufficient for the prespecified multivariable model, we will reduce model complexity by prioritizing predefined core predictors and, if necessary, use penalized regression approaches to improve model stability.

### 3.7 Missing data handling and internal validation

Missingness is summarized by variable. The primary outcome is not imputed; however, the outcome is included as a predictor in the imputation models for covariates/exposures. Multiple imputation by chained equations (MICE) is used for covariates (and for exposures if missingness is non-negligible and plausibly missing at random), with the outcome included in the imputation model. Primary analyses report pooled estimates from imputed datasets, while complete-case analyses are presented as sensitivity analyses.

If missingness is high, we may simplify the model or transparently report limitations.

To assess model robustness and avoid overfitting in multimodal classification models for identifying PHQ-9 ≥ 10 at baseline, internal validation is performed using bootstrapping (e.g., 500–1,000 resamples) to obtain optimism-corrected performance metrics (AUC, calibration slope/intercept, Brier score). Corrected AUC values and 95% CIs are calculated, and model performance variability across samples is examined. These results help evaluate the generalizability of the model and will be discussed in the final manuscript. Sample size is driven by model stability and planned internal validation. Assuming ∼15%–25% prevalence of PHQ-9 ≥ 10, the expected number of events will support parsimonious models with prespecified key predictors, with shrinkage/internal validation to mitigate overfitting.

### 3.8 Data management and confidentiality

All participant data are de-identified and assigned a unique study ID. The key linking personal identifiers to study IDs is stored separately and securely at each local site, accessible only to authorized site investigators. Electronic Data Capture (EDC) systems with password protection and audit trails are used for data entry. Data transmission between centers and the lead center is encrypted. All paper-based records (e.g., informed consent forms) are stored in locked cabinets at participating sites. Data quality checks (e.g., range checks, logic consistency) are performed regularly by the data management team at the lead center.

### 3.9 Ethics and dissemination

At the time of submission, ethical approval has been obtained from the Ethics Committee of the China Academy of Chinese Medical Sciences (No. 2025-EC-KY-004) and Hengshui Hospital of Traditional Chinese Medicine (No. 2025-KY-18). Recruitment at any additional participating site will commence only after site-specific ethical approval and local authorization have been completed. The study will be conducted in accordance with the Declaration of Helsinki. All participants must provide written informed consent before enrollment. The results of this study will be disseminated through peer-reviewed publications and conference presentations.

## 4 Planned presentation of results

As this is a study protocol, no results are reported. This section briefly outlines the prespecified analytic domains and the general approach to presenting the study findings after study completion.

### 4.1 Baseline characteristics

Baseline characteristics of the enrolled CAG cohort will be summarized overall and by PHQ-9 screening status (<10 vs. ≥10). These descriptive data will include demographic characteristics, lifestyle factors, comorbidities, histological and endoscopic severity, and questionnaire-based symptom measures. Baseline comparisons will be presented primarily descriptively, with standardized mean differences used where appropriate to characterize between-group imbalance.

### 4.2 Descriptive comparison of multimodal measures by PHQ-9 screening status

Multimodal measures will be described according to PHQ-9 screening status. These measures will include dorsalis pedis ultrasound indices, arterial stiffness and peripheral vascular indices, autonomic parameters, and questionnaire-based symptom measures. The primary descriptive comparison will remain PHQ-9 < 10 versus PHQ-9 ≥ 10. Additional analyses across broader PHQ-9 severity categories may be presented as exploratory analyses when appropriate.

### 4.3 Prespecified multivariable association analyses

Prespecified multivariable analyses will examine the associations between vascular and autonomic measures and depressive symptom burden. The primary model will use PHQ-9 ≥ 10 as the binary outcome in logistic regression. Secondary analyses may additionally examine PHQ-9 total score as a continuous outcome. Effect estimates will be reported with corresponding confidence intervals, with interpretation focused on effect size, precision, and consistency rather than on isolated statistical significance.

### 4.4 Internal evaluation of prespecified models

Prespecified models incorporating clinical, vascular, and autonomic variables will be internally evaluated for their ability to identify PHQ-9 screening-positive depressive symptom burden. Discrimination and calibration measures may be summarized to describe model behavior within the study sample, with internal validation used to limit overinterpretation. Any threshold-based summaries will be treated as exploratory and will not be interpreted as ready for immediate clinical implementation.

## 5 Discussion

### 5.1 Principal findings and study contributions

The main contribution of this protocol is to examine whether vascular and autonomic measures may serve as adjunctive markers of depressive symptom burden in a routine gastroenterology setting, particularly among patients who primarily present with somatic complaints. Rather than replacing standard psychological assessment, the study is intended to evaluate whether these physiological measures may provide additional clinically interpretable information within a histologically defined CAG population.

Previous research has consistently demonstrated a stable comorbidity pattern between upper gastrointestinal (GI) disorders—such as gastritis and functional dyspepsia—and psychological symptoms, including depression and anxiety. A network analysis by Tang et al. (21) in patients with chronic gastritis revealed that anxiety and depressive symptoms often cluster as core nodes within symptom networks, closely connected to multiple gastric complaints. Within this broader spectrum of chronic gastritis, CAG represents a precancerous subtype that may exhibit particularly persistent symptoms and psychosomatic burden, which motivates the present CAG-focused protocol. This supports the view that the “gastric–emotion” relationship is not merely incidental, but instead represents a structured psychosomatic coupling. Furthermore, large-scale Mendelian randomization studies have provided bidirectional causal evidence between depression and various GI diseases, lending genetic-epidemiological support to the concept of a two-way “emotion–gut” interaction (22, 23).

Building upon this foundation, the core hypothesis of our study is that hemodynamic characteristics of the dorsalis pedis artery and systemic arterial stiffness parameters are independently associated with depressive symptom burden in patients with CAG, a precancerous subtype of chronic gastritis, within a broader exploratory multimodal framework involving gastrointestinal, vascular, autonomic, and symptom-related measures. Recent research on vascular dysfunction has expanded beyond large-vessel stiffness to include microvascular abnormalities. Prior studies have reported associations between macrovascular or microvascular impairment and depressive symptoms in some populations (20, 24, 25), although these findings do not establish the specific mechanistic role of dorsalis pedis hemodynamics in the present study setting.

If observed, such associations would be interpreted as cross-sectional correlates within this multimodal framework rather than as evidence of a specific mechanistic pathway, and could help inform future longitudinal validation.

Separately, studies by Wang et al. (26), Galin et al. (27) have consistently shown that patients with depression exhibit reduced heart rate variability (HRV), with HRV indices inversely correlated with depression severity, indicating a significant role for autonomic imbalance in the pathogenesis of depression (28). Our proposed study will jointly analyze HRV metrics, arterial function, gastritis pathology, and depressive symptoms. If these findings are replicated, and if model performance improves after the addition of vascular and autonomic variables beyond clinical and gastrointestinal measures alone, this would support further evaluation of a multimodal assessment framework in future studies.

It must be emphasized that this is an exploratory observational study. Any findings, if validated, are intended primarily to provide a preliminary multimodal evaluation framework and a basis for hypothesis generation, rather than definitive conclusions about causality.

### 5.2 Clinical implications for digestive practice

Depressive and anxiety symptoms are not only common among patients with GI disorders, but are also associated with longer hospital stays, increased healthcare costs, and higher complication risks. However, in populations with gastritis or functional dyspepsia, emotional symptoms are often regarded by both patients and clinicians as secondary issues, and therefore remain insufficiently assessed (29, 30). In addition, symptom presentation in atrophic gastritis may itself be heterogeneous, and dyspeptic complaints do not always correspond in a simple way to underlying pathological severity, which further supports cautious clinical characterization in this setting (31).

If the observed associations are supported, the findings may help inform future efforts to integrate physiological and questionnaire-based assessment in gastroenterology settings. For patients who primarily present with somatic complaints, vascular and autonomic measures—when interpreted alongside symptom questionnaires—may provide additional clinically interpretable information for further evaluation, while remaining insufficient as stand-alone indicators of depressive disorder. Any future use in referral-oriented workflows would require external validation, clearer reference standards, and evaluation in real-world implementation studies.

Such an integrated assessment approach may be relevant to broader efforts to incorporate mental health assessment into the care of physical illnesses, although its practical value would still require external validation.

### 5.3 Integration with TCM perspectives

TCM has historically emphasized links between gastrointestinal function and emotional wellbeing. In this protocol, these perspectives are included only as brief contextual background. The study’s primary contribution remains the evaluation of a standardized multimodal assessment strategy in gastroenterology settings.

### 5.4 Future directions

Future studies should include external validation, longitudinal evaluation, and formal assessment of implementation and referral outcomes across a broader range of gastroenterology settings.

### 5.5 Limitations and conclusion

Several inherent limitations should be acknowledged. First, the proposed design is a multicenter, cross-sectional observational study conducted at specialist hospitals in northern China, which—even with adequate sample size—limits the ability to infer temporal or causal relationships between gastritis severity, vascular dysfunction, and depression onset. Observed associations should be interpreted as co-occurring patterns or cross-sectional correlates, not mechanistic pathways. Consistent with this cross-sectional design, all uses of terms such as “screening,” “risk stratification,” or “prediction” in this protocol refer to the cross-sectional identification of patients with higher concurrent depressive symptom burden, rather than forecasting future onset of depression. Although cross-sectional data cannot establish temporal direction or causality, they are appropriate for the present study’s aims of phenotype characterization, association analysis, and concurrent identification of questionnaire-defined symptom burden within a defined clinical population. Any future referral-related application would require more than a cross-sectional statistical association and should be evaluated only after external validation and clearer clinical reference standards. Referral uptake reflects the combined screening-and-communication process and cannot be attributed solely to the vascular findings themselves.

Second, the dorsalis pedis artery is only one of many assessable peripheral arteries; whether its hemodynamic alterations accurately reflect systemic microvascular status remains uncertain. Future studies should integrate retinal and cerebral microvascular imaging for triangulation.

Third, although this is a multicenter study, all participating hospitals are specialist centers located in northern and eastern China, which implies potential limitations in generalizability, related to patient characteristics, workflow, and equipment availability, and external validation in diverse healthcare settings will be necessary. In addition, cultural factors related to stigma, symptom reporting, and willingness to accept mental health referral may differ across settings, which may further limit direct extrapolation to other populations and healthcare systems.

Fourth, because all participants are patients with histologically confirmed CAG, the observed associations primarily describe within-CAG heterogeneity and should not be extrapolated to non-atrophic gastritis or the general population or to individuals with depression in the absence of gastritis. This caution is also consistent with prior literature showing that chronic atrophic or autoimmune gastritis may present with clinically heterogeneous manifestations and therefore requires interpretation within a clearly defined disease context (32). In addition, multicenter evidence from autoimmune atrophic gastritis has shown that clinically relevant extra-gastric manifestations, including hematologic abnormalities, may coexist at diagnosis, further underscoring the heterogeneity of this disease spectrum (33). Moreover, eligibility required recent upper gastrointestinal endoscopy and histological assessment, which may preferentially include patients with more persistent or bothersome symptoms and higher care-seeking behavior. As a result, the findings may not fully generalize to community-dwelling individuals with milder or undiagnosed gastritis. In addition, although we followed the EPV ≥ 10 rule for sample size planning and adopted internal validation to mitigate overfitting, the number of candidate predictors remains high, which could inflate model complexity. External replication in independent cohorts will be essential.

Despite these limitations, the study may offer methodological and clinical value. By integrating dorsalis pedis ultrasound, arterial stiffness measures, heart rate variability, histological gastritis staging, and validated scales for depression, anxiety, and sleep, this protocol provides a framework for evaluating whether multimodal physiological measures may assist with the concurrent identification of depressive symptom burden in CAG. If the observed associations are supported and later replicated, they may inform future validation studies and more formal evaluation of practice-based assessment pathways in gastroenterology care. External validation across diverse regions and clinic levels will be necessary to evaluate generalizability and equity of implementation.

Residual confounding related to medication exposure, autonomic state, recent behavior, and other unmeasured physiological influences cannot be fully excluded.
